# An Evaluation of Boar Spermatozoa as a Biosensor for the Detection of Sublethal and Lethal Toxicity

**DOI:** 10.3390/toxins10110463

**Published:** 2018-11-08

**Authors:** Emmanuelle Castagnoli, Johanna Salo, Matti S. Toivonen, Tamás Marik, Raimo Mikkola, László Kredics, Alejandro Vicente-Carrillo, Szabolcs Nagy, Markus T. Andersson, Maria A. Andersson, Jarek Kurnitski, Heidi Salonen

**Affiliations:** 1 Department of Civil Engineering, Aalto University, Rakentajanaukio 4, 02150 Espoo, Finland; johanna.72salo@gmail.com (J.S.); raimo.mikkola@aalto.fi (R.M.); maria.a.andersson@helsinki.fi (M.A.A.); jarek.kurnitski@ttu.ee (J.K.); heidi.salonen@aalto.fi (H.S.); 2 Department of Applied Physics, Aalto University, Puumiehenkuja 2, 02150 Espoo, Finland; toivosenmatti@gmail.com; 3 Faculty of Science and Informatics, Department of Microbiology, University of Szeged, Közép Fasor 52, H-6726 Szeged, Hungary; mariktamas88@gmail.com (T.M.); kredics@bio.u-szeged.hu (L.K.); 4 Evidensia Valla Djursjukhus Linköping, Westmansgatan 21, 58216 Linköping, Sweden; alejandro.vicentecarrillo@gmail.com; 5 Georgikon Faculty, Department of Animal Sciences, University of Pannonia, Deak F. u. 16, H-8360 Keszthely, Hungary; nagy.szabolcs@georgikon.hu; 6 Independent Scholar, 00550 Helsinki, Finland; markus.t.andersson@gmail.com; 7 Department of Civil Engineering and Architecture, Tallinn University of Technology, Ehitajate tee 5, 19086 Tallinn, Estonia

**Keywords:** biological toxins, biosensor, boar spermatozoa, MATLAB, toxicity, computed motility inhibition, CASA

## Abstract

A novel, objective, and rapid computed motility inhibition (CMI) assay was developed to identify and assess sublethal injury in toxin-exposed boar spermatozoa and compared with a subjective visual motility inhibition (VMI) assay. The CMI values were calculated from digital micrographic videos using a custom MATLAB^®^ script by contrasting the motility index values of each experiment with those of the background and control experiments. Following a comparison of the CMI and VMI assays results, it was determined that their agreement depended on the shape of the dose-response curve. Toxins that exhibited a steep slope were indicative of good agreement between the assays. Those depicted by a gentle decline in the slope of the dose-response curve, the CMI assay were shown to be two times more sensitive than the VMI assay. The CMI assay was highly sensitive to the inhibition of mitochondrial function and glucose transport activity by sublethal doses of toxins and to disruption of cellular cation homeostasis by carrier ionophoric toxins, when compared to the cytotoxicity and lethal toxicity assays (i.e., that evaluated the inhibition of cell proliferation in somatic cell lines (FL, PK-15, and MNA cells)) and disruption to spermatozoa membrane integrity. The CMI assay recognized subtle sublethal toxicity changes in metabolism, manifested as a decrease in boar spermatozoa motility. Thus, it was feasible to effectively compare the objectively-measured numerical values for motility inhibition using the CMI assay against those reflecting lethal damage in the spermatozoa cells and somatic cell lines using a cytotoxicity assay.

## 1. Introduction

This paper evaluates the suitability of boar-spermatozoa bioassays in assessing the toxicity of selected biological toxins. The assessed boar spermatozoa (commercially available liquid extended boar semen) bioassays included newly developed computed motility inhibition (CMI), visual motility inhibition (VMI), boar spermatozoa motility inhibition (BSMI) and spermatozoa membrane integrity disruption (SMID) assays. The CMI, VMI, and BSMI [1] assays detect changes in the motility of toxin-exposed boar spermatozoa (sublethal toxicity), while the SMID assay [2] identifies damage to the plasma membrane (lethal toxicity) of toxin-exposed boar spermatozoa.

The motility of boar spermatozoa can be reversibly induced by warming it to 37 °C in the presence of oxygen or turned off by cooling it to 15–18 °C and via oxygen depletion [3]. BSMI has been employed as a model system for screening, identifying, or excluding mitochondrial and membrane toxicity in drugs, and microbial contaminants in food and indoor environments [4,5,6,7,8,9,10,11]. Assessment of spermatozoa motility inhibition has been objectively carried out using semiautomated computer-aided sperm analysis (CASA) [7,8,9,10,11,12], the semiautomated QualiSperm^TM^ motility analyzing systems [11], subjective visual evaluation of the inhibition of boar spermatozoa motility under phase-contrast microscopy, BSMI assay, or a combination of these methods [1,7,8,9,10,11,12]. Even though visual subjective and semiautomatic objective assessments of the inhibition of spermatozoa motility are strongly related, distinctive and differing methods are employed to measure motility inhibition [13]. The main disadvantage of CASA analysis [14], and the QualiSperm^TM^ motility analyzing system is the necessity to purchase expensive equipment.

The BSMI assay is a rapid and cost-effective method based on visual inspection, for which tail beating is applied as the criterion when estimating the rate of progressive rapid motility [1]. However, the inability of a trained human eye to recognize subtle changes in spermatozoa motility prevents the acquisition of quantitative dose-response curves reflective of a decrease in spermatozoa motility. An escalation in motility is also impossible to quantify with the BSMI assay. The subjectivity of the visual assessment of spermatozoa motility is a well-known disadvantage of this method [13]. Under commercial conditions, where economic concerns are important [13], and particularly in enterprises providing threshold values for risk assessment, the inhibition of spermatozoa motility should be assessed using objective and subjective methods. Accordingly, it was determined that the use of an objective method to measure motility inhibition, while utilizing the same technical equipment in conjunction with visual inspection, would be a useful alternative to measuring the inhibition of motility in the exposed cells.

To assess spermatozoa motility in animal breeding, the mass activity of the samples must be visually inspected under a microscope [15,16]. Mass activity is a function of spermatozoa motility and spermatozoa concentration. Mass action or “swirl” can be observed in a droplet inspected without a coverslip with 100× magnification and the condenser properly adjusted. Here, the mass action of exposed spermatozoa cells can be used as a measure of motility. In the present study, a simple, objective, and rapid method of obtaining results was developed using a MATLAB^®^ algorithm to compute a motility inhibition index for toxin-exposed boar spermatozoa. Agreement with the objective CMI index in the CMI assay, and the inhibition index obtained by visual inspection in the VMI assay, simultaneously recorded from the same microscopic video frames, was evaluated. The readouts of the CMI were compared to those obtained with the cytotoxicity assays performed on boar spermatozoa and inhibition of proliferation in somatic cell lines. The application of CMI and VMI to rapidly screen for toxigenic microbial single colonies is described.

## 2. Results

### 2.1. Motility Inhibition of Toxin-Exposed Boar Spermatozoa Recorded by CMI, VMI, and BSMI Assays

The sensitivity of the CMI assay was evaluated by comparing its half maximal effective concentration (EC_50_) to the ones obtained with the VMI and BSMI assays. Here, the EC_50_ represented the concentration at which the toxin-exposed spermatozoa showed a 50% decrease in motility compared to the control.

**Figure 1:**
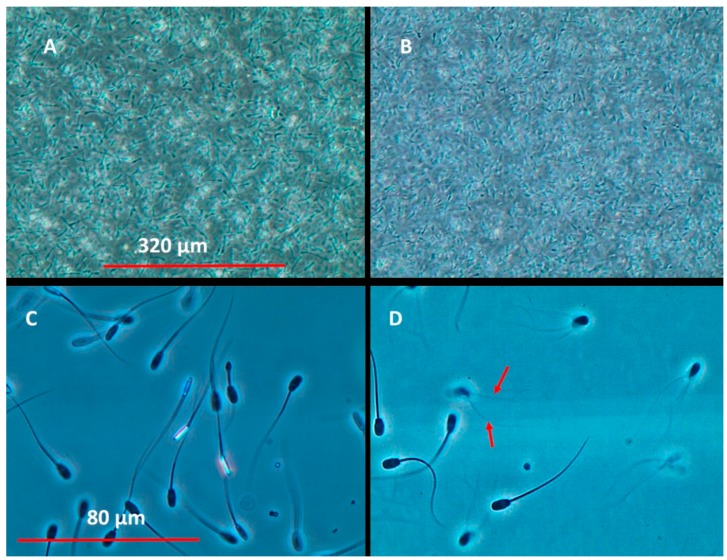
An example of the recorded video frames using phase contrast microscopy to measure the inhibition motility of toxin-exposed boar spermatozoa in the (**A**) VMI assay; (**B**) CMI assay, and (**C**,**D**) BSMI assay, the red arrows show optical artifact of a spermatozoa cell having two tails.

EC_50_ values in the VMI, CMI, and BSMI assays were determined from micrographic videos recorded using a phase contrast microscope (Olympus CKX41, Tokyo, Japan). Differences in the magnification and spermatozoa counts used for motility inhibition measurements in the three spermatozoa assays are highlighted in Figure 1. To avoid unconscious bias, the VMI and BSMI assays were consistently performed prior to the CMI assay.

The EC_50_ concentration values obtained for the microbial toxins (alamethicin, calcimycin, chaetoglobosin A, and valinomycin) and the anthropogenic toxins (carbonyl cyanide 4-(trifluoromethoxy) phenylhydrazone (FCCP), and triclosan) using the VMI, CMI, and BSMI assays are summarized in Table 1.

**Figure 2:**
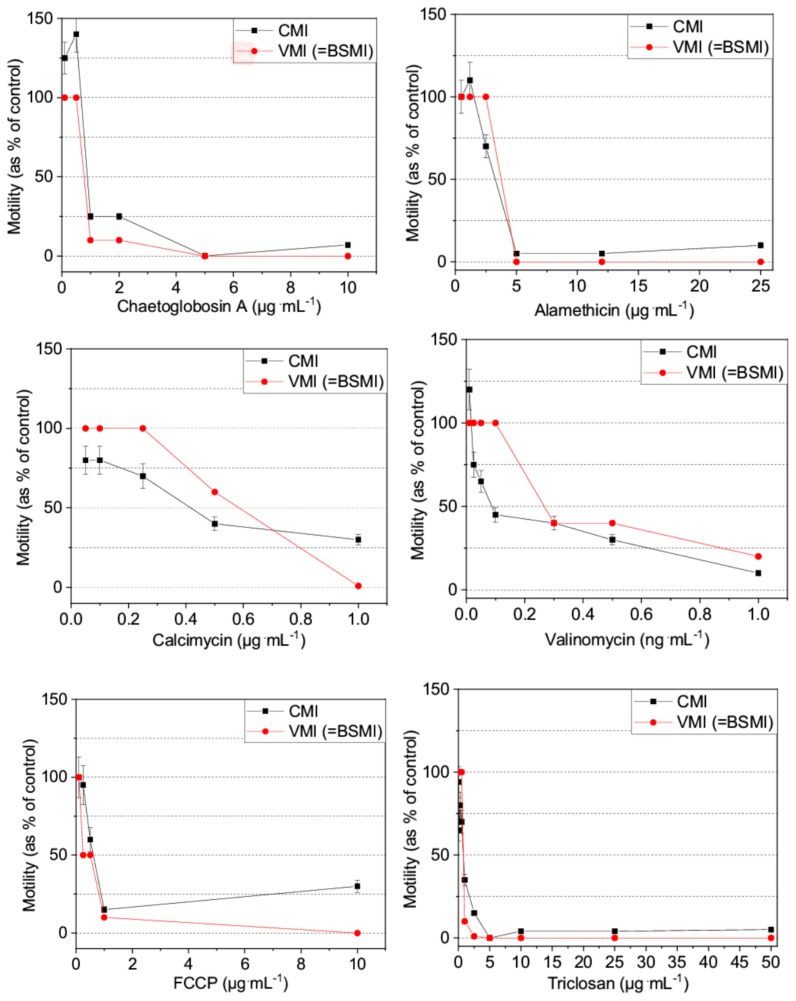
Dose-response curves for the inhibition of motility in toxin-exposed boar spermatozoa cells by visual inspection (i.e., VMI assay; the results of which were identical to those of the BSMI assay) and computed with a MATLAB^®^ algorithm (CMI assay) from the same video recordings. EC_50_ concentrations are listed in Table 1.

The dose-response curves reflective of toxicity (Table 1) showed that identical EC_50_ end-points were obtained for certain toxins (i.e., alamethicin, triclosan, FCCP, and chaetoglobosin A) depicted by a steep descent in spermatozoa motility in a narrow range of concentration (Figure 2). The dose-response curves obtained using the VMI and BSMI assays were identical.

The EC_50_ end-points for valinomycin and calcimycin, two carrier ionophores that affect the mitochondria, were 2–3 times higher using the VMI and BSMI assays, compared to the CMI assay because their dose-response curves exhibited a slow decrease in spermatozoa motility across a broad concentration range.

Interestingly, only the CMI assay detected an increase in motility after exposure to low subtoxic concentrations of alamethicin (<2.5 µg·mL^−1^), chaetoglobosin A (<1 µg·mL^−1^) and valinomycin (<0.1 ng·mL^−1^). The CMI assay also detected that high concentrations of alamethicin (25 µg·mL^−1^), chaetoglobosin A (10 µg·mL^−1^) and FCCP (10 µg·mL^−1^), provoked reduced motility inhibition compared to that provoked by more diluted concentrations (Figure 2). The comparative results obtained using the VMI and BSMI assays supported the validity of the CMI assay and also demonstrated that the latter had greater sensitivity in being able to detect subtle and unexpected changes in the dose-response of motility inhibition incited by certain toxins.

The VMI and CMI assays were recorded without a coverslip using 100× magnification (10× eyepiece and 10× objective). The optical signal was provided by >10,000 spermatozoa cells. (A) Double-frozen spermatozoa cells representing the complete inhibition of motility (0%) and depicting the optical background signal needed to compute the inhibition of motility. (B) Motile spermatozoa cells with a CMI value of >70% and a VMI assay value of 100%. The BSMI assay video frames were recorded from coverslip samples representing ca. 10–30 spermatozoa cells per frame using 400× magnification (10× eyepiece and 40× objective). (C) Double-frozen spermatozoa with 0% motility and (D) spermatozoa cells exhibiting rapid tail beating visualized as an optical artifact of spermatozoa cells having two tails (red arrows) in 70% of cells.

### 2.2. Toxins Detected Using the CMI and SMID Assays

The specificity of the CMI assay was evaluated against disruptions to the integrity of the spermatozoa membrane using the SMID assay and the inhibition of cell proliferation (ICP) assays based on the responses of feline fetus lung (FL) cells, murine neuroblastoma (MNA) cells and porcine kidney (PK-15) cells to selected fungal toxins (mycotoxins), bacterial toxins, and toxic chemicals (Table 2). The toxins were selected based on knowledge of their known biological targets and the absence of knowledge regarding unknown targets, e.g., ochratoxin and citrinin, in *in vitro* assays. Ochratoxin A constituted the negative control (the absence of a toxic response).

Four different type of toxic responses were observed:

- Response I: The lowest toxic response identified using the CMI assay (to chaetoglobosin A, calcimycin and valinomycin)
- Response II: The lowest toxic response identified using both the CMI and SMID assays (to FCCP and triclosan)
- Response III: The lowest toxic response in the SMID assay (to alamethicin)
- Response IV: The toxic responses in the ICP assays only (to sterigmatocystin, citrinin, actinomycin D and potassium dichromate).

The spermatozoa-based assays (CMI and/or SMID assays) detected toxins that inhibited mitochondrial functions and glucose transport activity, as well as those that disrupted cellular cation homeostasis, but were insensitive to toxins that affect nucleic acid and protein synthesis.

The CMI assay was more sensitive and quicker (exposure of only 20 min) in identifying mitochondrial and carrier ionophoric toxins (responses I, II, and III) than the ICP assay (exposure of 1–3 days).

Interestingly the CMI assay was also sensitive to chaetoglobosin A, a glucose transport inhibitor.

Having established the EC_50_ values objectively using the CMI assay, it was possible to correlate the spermatozoa motility inhibition values with those obtained for plasma membrane-related damage to toxin-exposed spermatozoa cells measured using the SMID assay. The CMI spermatozoa assay was more sensitive and faster (exposure of only 20 min) in detecting chaetoglobosin A, calcimycin, and valinomycin (responses I and II) toxins than the SMID assay (exposure of two hours). However, the lowest toxicity levels for chaetoglobosin A, calcimycin, and valinomycin were detected with the SMID assay which had six times longer incubation period than the CMI assay. Thus, this indicates that these toxins were not noxious to the spermatozoa cells, in contrast to alamethicin, which was shown to deplete the integrity of the plasma membrane.

Both the CMI and SMID spermatozoa assays were insensitive to known inhibitors of macromolecular synthesis, i.e., sterigmatocystin and actinomycin D.

### 2.3. Applicability of the CMI Assay in Screening the Toxicity of Microbial Colonies Collected from Environmentally Compromised Buildings

**Figure 3:**
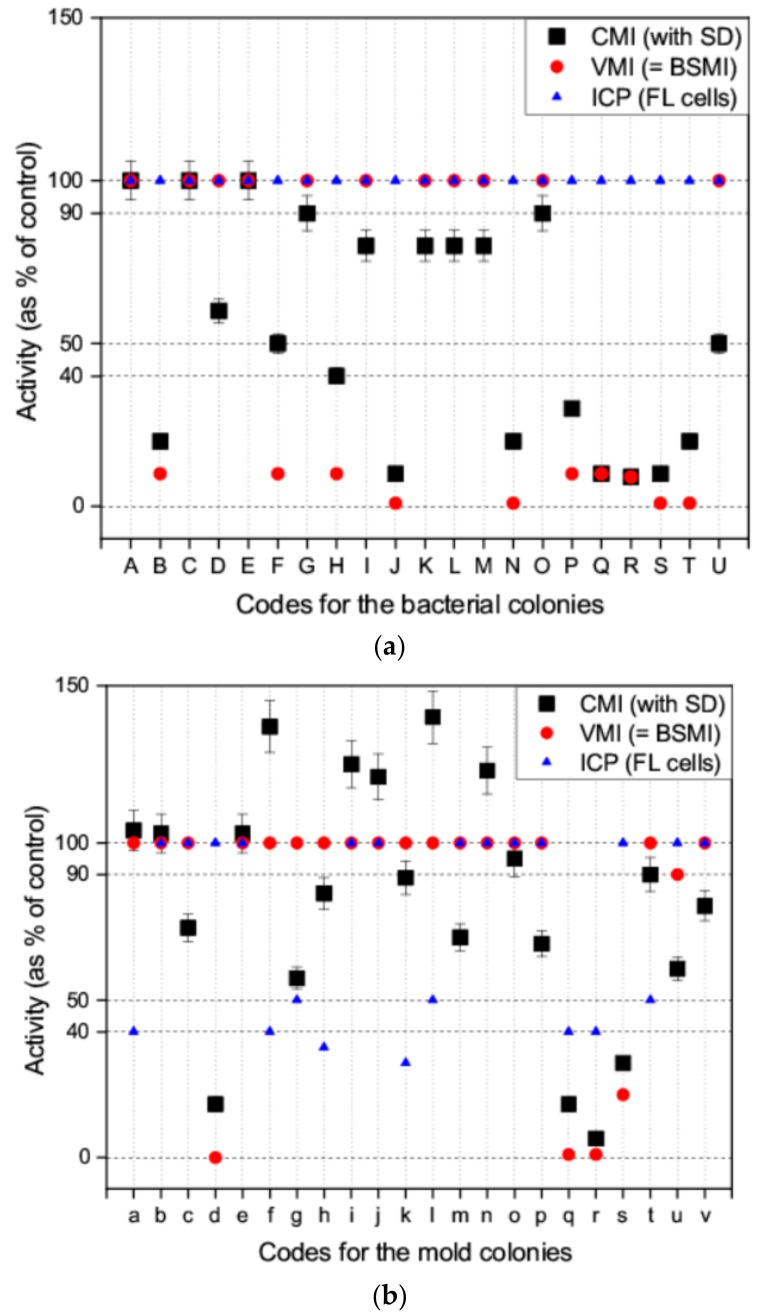
Responses to the toxins measured as a decrease in activity provoked by bacterial colonies (**a**) and mold colonies (**b**) and analyzed using four toxicity assays. The VMI and BSMI assays (red circle) demonstrated the inhibition of spermatozoa motility, subjectively by visual inspection. The red circles indicate the findings of the VMI and BSMI assays (identical results). The CMI assay (black square) shows the objective measurement of the inhibition of spermatozoa motility using a computed MATLAB^®^ algorithm. The mean of the three parallel measurements is shown; the SD is depicted as an error bar. The ICP assay (blue triangle) shows the inhibition of cell proliferation in the somatic FL cell lines. The decrease in motility was estimated from four digital videos. The mean of the three measurements is shown (SD was <20%).

Applicability of the new computer-based assay (CMI assay) was tested to screen the toxicity of microbial colonies collected in environmentally compromised indoor environments. In total, 21 bacterial colonies (Figure 3a) and 22 mold colonies (Figure 3b) were collected from buildings with reported indoor air problems. These microbial colonies produced bioactive substances affecting spermatozoa motility, that is, mitochondrial functions, cellular ion fluxes, and energy metabolism.

Ten of the bacterial colonies did not provoke a toxic response in the exposed spermatozoa cells when evaluated using the VMI assay (spermatozoa motility was shown to be similar or almost similar to that of the control) but activity of >50% was revealed using the CMI assay (Figure 3a). Nine of the bacterial colonies were shown to reduce spermatozoa motility to <40% using the VMI and CMI assays. The spermatozoa motility of colonies F and U was estimated to be 50% using the CMI assay, but was visually interpreted as 10% for colony F and 100% for colony U (using the VMI assay and BSMI assay). None of the bacterial colonies were shown to inhibit the proliferation of exposed FL cells using the ICP assay. The results obtained with the VMI, BSMI and CMI assays correlated for 90% of the colonies (19/21 colonies). The human eye (VMI assay) assessed motility of >60% (CMI assay) to be equal to that of the control. Motilities computed <40% (CMI assay) were recognized by the human eye as >50% motility inhibition (VMI assay). Motility of 50% using the CMI assay could not be accurately assessed visually.

Eighteen of the mold colonies (biomass suspension) did not provoke a toxic response in the exposed spermatozoa cells. The degree of motility estimated using the VMI and BSMI assays was similar to that for the control and was seen to be >50% with the CMI assay (Figure 3b). Spermatozoa motility was seen to decrease to <40% in four of the mold colonies (d, q, r, and s) using the VMI and CMI assays (Figure 3b). The results obtained with the VMI, BSMI, and CMI assays correlated with each one of the mold colonies assessed. An increase in spermatozoa motility was demonstrated in mold colonies f, i, j, l, and n using the CMI assay only. This increase could not be observed visually. This suggests that the CMI assay was capable of detecting subtle changes in cell metabolism and behavior, that manifested as spermatozoa cell motility.

The biomass suspensions of mold colonies d and s inhibited spermatozoa motility (VMI, BSMI and CMI assays) but not cell proliferation (ICP assay). This suggests a significant degree of insensitivity with the use of the ICP assay in the detection of certain toxins, in contrast to the VMI, BSMI, and CMI assays. Mold colonies q, and r inhibited both spermatozoa motility (VMI, BSMI and CMI assays) and cell proliferation (ICP assay). Nine of the mold colonies (a, f, g, h, k, l, q, r, and t) inhibited cell proliferation (ICP assay) but only colonies q and r also demonstrated a decrease in motility in the exposed spermatozoa (Figure 3b).

Mold colonies q and r, identified as toxic in all the assays (CMI, VMI, BSMI, and ICP), were pure cultured and identified as trichorzianine-producing *Trichoderma atroviride* (strains 14/AM) and ophiobolin H-producing *Aspergillus calidoustus* (strain MH34), respectively. Mold colonies d and s were observed to be toxic to the spermatozoa cells only and were identified as viriditoxin-producing *Paecilomyces* *variotii* (strains Paec 1 and Paec 2). The following colonies, identified as toxic in the ICP assay only, were identified as chaetoglobosin- and communesin-producing *Penicillium* *expansum* (RcP61, Colony a); meleagrin-producing *P. chrysogenum* (RUK/2, Colony f); sterigmatocystin- and averufin-producing *Asp. versicolor* SL/3 (Colony g) and melinacidin-producing *Acr. luteoalbus* (POB8, Colony k) (Figure 3b). Mold colonies, h, l, and t, were not identified.

These results indicate that 50% of the tested bacterial colonies produced spermatozoa motility-inhibiting toxins, whereas only 5% of the mold colonies inhibited spermatozoa motility (Figure 3). This indicates that toxins that affect the mitochondria, cellular ion fluxes, and energy metabolism are frequently produced by bacteria.

## 3. Discussion

The advantages of using boar spermatozoa as a biosensor for sublethal and lethal toxicity were evaluated in this paper. The classic BSMI assay was evaluated against the CASA motility evaluating system described by Bencsik et al. [1] and Andersson et al. [7], and developed into a new CMI assay that objectively determines boar spermatozoa motility inhibition. The CMI assay uses a decrease in the motility index, computed using a MATLAB^®^ algorithm that correlates with the inhibition of spermatozoa motility in the toxin-exposed spermatozoa cells, as a measure of toxicity. Computed motility inhibition (CMI) was calculated based on collective motility, similar to the mass activity of the spermatozoa cells in the sample recorded in a digital video. The same digital videos were also analyzed by visual inspection to subjectively estimate the inhibition of spermatozoa motility using the VMI and BSMI assays.

The sensitivity of the CMI assay was evaluated against that of the VMI and BSMI assays. A comparison of the EC_50_ values obtained with the CMI, VMI, and BSMI assays revealed that their agreement depended on the shape of the dose-response curve. Good agreement was obtained for toxins, like alamethicin, that were depicted using a steep slope. The steep slope that is representative of chaetoglobosin A, can be elucidated by the inhibition of sugar transport activity, thereby causing depletion in the production of glycolytic and mitochondrial energy [18].

The EC_50_ value of valinomycin (a mitochondrial toxin) in the CMI assay [17], was three times smaller than that obtained using the VMI assay, and can be explained by the slow descent in the dose-response curve, indicating that the CMI assay was able to detect subtle differences in motility that were imperceptible using VMI assay (i.e., visual estimation with the human eye). This is supported by the observation that the CMI assay (but not the VMI or BSMI assays) recorded an increase in motility, compared to the control, after exposure to low subtoxic concentrations of alamethicin, chaetoglobosin A, and valinomycin, and following exposure to the biomass suspensions of five of the mold colonies (f, i, j, l and n). Thus it was possible to detect subtle changes in metabolism and behavior manifested in the motility of spermatozoa cells using the CMI technique. Elsewhere, increased sperm motility was incited by calcium ionophore and caffeine [20,21], detected using computer-based analysis [22]. The results obtained with the CMI assay also indicated that high concentrations of alamethicin, chaetoglobosin A, and FCCP provoked a reduction in the inhibition of spermatozoa motility, compared to more diluted concentrations. A reduction in the inhibition of spermatozoa motility may have been caused by the reduced bioavailability of the toxin due to micelle formation. This result illustrates the shortcomings of the subjective techniques, like the VMI and BSMI assays, in recording unexpected results.

The CMI and VMI assays described in this study were based on an evaluation of the mass activity of spermatozoa cells, whereas the existing BSMI and CASA systems, typically used in toxicity assays, are generally used to estimate the percentage of individual motile spermatozoa [7,12]. When applied to assess toxicity, the subjective assay (VMI and BSMI assays) results were confirmed by the objective newly developed, computer-based assay (CMI assay). Compared to the control, a decrease in motility of <40% was correctly identified by the human eye, while the identification of motility of ≥60% was similar to that of the control. However, when the decrease in motility was exactly 50%, it was difficult to grade and estimate using the human eye.

The specificity of the CMI assay was tested against that of the SMID assay (used to detect disruptions to the integrity of the spermatozoa membrane) and the ICP assay (used to assess the inhibition of cell proliferation in somatic cells (PK-15, MNA, and FL cells)). Overall, the spermatozoa-based assays (CMI and SMID assays) were similarly sensitive to toxins inhibiting mitochondrial functions, glucose transport activity, and that disrupted cellular cation homeostasis. Yet, the CMI assay displayed higher sensitivity than SMID and ICP assays to toxins such as valinomycin. Both spermatozoa-based assays were insensitive to toxins that affected nucleic acid and protein synthesis and that the ICP assay was able to detect. The CMI assay only required 20 min of exposure to the toxins.

Boar spermatozoa motility inhibition has been shown to be instrumental when screening for toxigenic microbes and in the purification and identification of their isolated toxins. In combination with motility inhibition, functional staining of mitochondria and plasma membrane, as well as measurements of cellular ATP and NADH contents in motile and immobilized spermatozoa, have revealed the biological target of new microbial toxins [5,6,8,9,11,23]. The battery of assays employing and comparing spermatozoa motility inhibition, disruption to the integrity of the spermatozoa plasma membrane, and the inhibition of cell proliferation distinguished carrier ionophoric toxins affecting mitochondria like valinomycin, sugar transport-inhibiting chaetoglobosin A, and ion channel formers like alamethicin.

When the findings of the fluorometric cytotoxicity assay (ICP) and the CMI and SMID spermatozoa assays were compared, the evaluated endospore-forming bacteria (*Streptomyces* spp.) produced spermatozoa mobility-inhibiting agents that did not affect the proliferation of somatic cell lines, indicating that these bacteria produce mitochondrial toxins similar to valinomycin, cereulide, and paenilide [24].

However, the following aspects should be considered when making a comparison between the findings of motility and membrane integrity assays. Most membrane integrity assays, such as SMID (using propidium iodide [PI]) is limited to evaluating the integrity of the plasma membrane status of the spermatozoa head only. Consequently, such assays tend to overestimate the percentage of intact, viable cells, compared to the motility assays [25].

In addition, assays that determine the integrity of the plasma membrane tend to produce a quantal response (i.e., viable or dead), whereas motility assays (especially computerized tests) indicate a quantitative response, thus requiring a different statistical approach to the data analysis [26]. Computer-assisted motility analysis has been shown to be a more robust tool for end-point determination in reproductive toxicology studies than membrane integrity assays, even using flow cytometry [27].

Mold colonies that provoke the inhibition of spermatozoa motility were identified as ophiobolin-producing *Asp. calidoustus*, trichorzianine-producing *T. atroviride* and viriditoxin-producing *Pae. variotii*. Trichorzianines are channel-forming peptaibols [28] and viriditoxin is a mitochondrial toxin [29], while the biological effects of ophiobolin H on the spermatozoa cells are not known [1]. Cytotoxic substances, such as sterigmatocystin (that inhibits protein synthesis; produced by colony g), meleagrin (produced by colony f), and melinacidin (produced by colony k) were not detected by the rapid spermatozoa assays, indicating that they did not affect mitochondrial functions, energy metabolism, nor ion homeostasis within 30 min to two hours of exposure. The subjective assays were suitably accurate for screening, purifying and identifying known and unknown toxic substances. Nevertheless, having access to objectively measured numerical values make it considerably easier to make the comparison of the findings with those of other assays.

## 4. Conclusions

Performing the described CMI assay is technically simple. Sophisticated equipment is not needed (i.e., it only requires an ordinary phase contrast microscope with a video camera) and a single measure is utilized to determine the inhibition of motility in toxin-exposed boar spermatozoa. Nevertheless, it requires promotion as an alternative to subjective, cost-effective motility inhibiting assays, such as the BSMI and VMI. Compared to the subjectivity that is inherent in these methods, by contrast the advantages of the new CMI assay are:

- It is able to recognize subtle changes in spermatozoa motility.
- It provides an objective motility inhibition value, thus facilitating the calculation of the SDs of measurements and accurate dose-response curves.
- It provides numerical values for an increase in spermatozoa motility compared to the control.
- Each measurement is independent of the previous one, thus, it is better equipped to identify contradictory and unexpected results
- It gives a numerical, computed value of the mass action of a high number of spermatozoa, i.e., >10 times the number measured by the BSMI or CASA systems.
- As the spermatozoa cells are inspected without the use of coverslip, the boar spermatozoa do not stick to the glass surface.

These characteristics make this assay useful for analyzing samples requiring risk assessments during environmental biomonitoring. Further research is in progress concerning the applicability of the CMI assay and its ability to measure motility in the spermatozoa cells of domestic animals and test organisms (i.e., nematodes, algae, and shrimps).

## 5. Materials and Methods

### 5.1. Experimental Design

An evaluation was conducted of the efficacy of the CMI assay in measuring a reduction in boar spermatozoa motility following exposure to a variety of toxic compounds. Validation of the CMI assay consisted of three steps:

- Sensitivity: Sensitivity was evaluated by comparing the EC_50_ concentration values obtained for the inhibition of motility in boar spermatozoa exposed to selected toxins determined by visual inspection (VMI assay) with those obtained for the inhibition of motility in boar spermatozoa using the BSMI assay.
- Specificity: Specificity of the response to the selected toxins was compared to the values obtained for the inhibition of motility in boar spermatozoa cells (SMID assay) and the inhibition of cell proliferation in the somatic cells (ICP assay).
- Applicability: The applicability of the rapid screening of toxic mold colonies was evaluated and compared to that of the VMI and ICP assays.

### 5.2. Indicator Cells

Boar semen (Figen Oy, Tuomikylä, Finland; density of 27 × 10^6^ cells mL^−1^) extended in MR-A (Kudus, Madrid, Spain) was used for the experiments within 2–4 days of collection. The semen batch was rejected if less than 80% of the spermatozoa cells were motile.

The porcine kidney (PK-15), murine neuroblastoma (MNA) and feline fetus lung (FL) cells were cultured in RPMI 1640 with L-glutamine, heat-inactivated fetal bovine serum albumin, and penicillin-streptomycin (10,000 units of penicillin and 10,000 μg·mL^−1^ of streptomycin) obtained from Gibco (Invitrogen, Carlsbad, CA, USA). The somatic cells were grown in a cell culture cabinet at 37 °C in a water-saturated atmosphere of 5% CO_2_ and 95% air.

### 5.3. Spermatozoa Motility Inhibition Assays (VMI, BSMI, CMI)

Extended boar semen aliquots (200 µL) were exposed to 0.5–2 µL of ethanol-soluble compounds. The exposed spermatozoa cells were incubated at 37 °C for 20 min.

Spermatozoa motility was determined from the edge of a magnified 10 µL semen drop (10× eyepiece and 10× objective) placed on a pre-warmed (37 °C) microscope slide examined with a phase contrast microscope equipped with a heated stage. The uncovered slip motile spermatozoa moved in and out of focus creating a rapid, distinct swirl. Six-second videos of the movement of semen were recorded using a digital camera (Olympus SC30, Tokyo, Japan) and a video-recording software (Cellsense^®^ standard version 11.0.06, Olympus Soft Imaging Solutions GmbH, Münster, Germany) having applied certain settings (exposure time 72 ms, resolution Live/Movie 680 × 512, Snap/Process 680 × 512 (binning 3)).

The maximal motility was calculated from videos of spermatozoa cells exposed to solvent only (negative control). The background value (positive control) was obtained from videos of completely immotile spermatozoa subjected to unprotected double freezing at −20 °C. The inhibition of motility in the spermatozoa mass was subjectively estimated (requiring the consensus of three operators) by visual inspection (VMI assay) and by computed motility index (CMI assay) using the same digital videos and 3–4 microscopic fields represented ca. 40,000 spermatozoa cells per evaluation.

In addition, the inhibition of motility in the individual spermatozoa was subjectively estimated (requiring the consensus of three operators) using the BSMI assay, and tail beating was a criterion for rapid and progressive motility, as described in Castagnoli et al. [2].

### 5.4. Calculation of the CMI Spermatozoa Index

Spermatozoa motility was calculated from the motility indices computed from digital micrographic videos using a customized MATLAB^®^ script, using a two-step process.

Firstly, the individual RGB frames were converted to intensity frames and processed as floating-point number matrices. The means and the standard deviation (SD) of these matrices were adjusted to 0 and 1, respectively, to minimize the effect of variation in total exposure between the video frames. This was achieved by subtracting the mean of the matrix from all its elements and subsequently dividing these values by the SDs obtained for elements of the matrix.

Secondly, the consecutive matrices were subtracted from one another, resulting in a “differential matrix” that highlighted the differences between the frames. The chosen motility measure was the mean of the absolute value of this differential matrix.

The motility indices used, and thus the spermatozoa motility reported in this work, were computed from the first 100 frames of each video. This number of frames was selected following an observation that ≤1% variation was typically reflected in the resulting motility index after the first 10 frames.

From these motility index values, the computed inhibition of motility in the spermatozoa was computed by contrasting the motility index of each experiment with the motility index values of the background and control (Equation (1)) as follows:

CMI (as % of control) = (MI_experiment_ − MI_background_)/(MI_control_ − MI_background_) × 100 (1)

where MI_experiment_ was the motility index of the experiment for which the spermatozoa motility was calculated; MI_background_ was the motility index of the positive control, that is, the double-frozen semen sample representing the background signal level deriving from the experimental setup; and MI_control_ was the motility index of the negative control experiment in which the spermatozoa sample was exposed to a small amount of ethanol only, without added solutes. The mean spermatozoa CMI was calculated with the Microsoft^®^ Excel^®^ 2013 (Microsoft Corporation, Redmond, WA, USA) function, AVERAGE. The SD was computed using Microsoft^®^ Excel^®^ 2007 function, STDEV. The assay was calibrated with triclosan (CAS: 3380-34-5; Sigma Chemical Co., St. Louis, MO, USA) using three parallel assays, performed with four different semen doses taken from four different boars. The EC_50_ of triclosan was 0.9 μg·mL^−1^ (SD ± 0.3).

A user-friendly, Windows^®^-based application was created to calculate the CMI index, based entirely on the algorithm previously described. This open source application was developed with C++. The code is included in the Supplementary Material (Software Source Code S1).

### 5.5. The SMID Assay

The assay that measures disruption to integrity of the cell membrane of motile spermatozoa cells was performed by PI staining (Molecular Probes, Eugene, OR, USA) as described by Castagnoli et al. [2]. The assay was calibrated with triclosan using five parallel tests (EC_50_ of 2 μg·mL^−1^; SD ± 0.6).

### 5.6. The ICP Assays

The inhibition of cell proliferation was tested in two continuous cell lines (PK-15 and FL cells) and one malignant cell line (MNA cells) according to the protocol of Bencsik et al. [1]. The assay was calibrated with triclosan using four parallel tests (EC_50_ of 8 µg·mL^−1^; SD ± 2).

### 5.7. The Preparation of Toxic Compounds for Toxicity Testing

Eleven toxic compounds (Sigma Aldrich, St. Louis, MO, USA) were purchased and tested:

- Actinomycin D A9415 (*Streptomyces* spp., CAS: 50-76-0, MW 1255.42)
- Alamethicin A4665 (*Trichoderma arundinaceum*, CAS: 27061-78-5; a mixture of Alamethicin F50 peptaibols, MW 1962, 1976, 1976 and 1990)
- Calcimycin A23187 (CAS: 52665-69-7 MW 523.62)
- Chaetoglobosin A (*Chaetomium globosum*, CAS: 50335-03-0 MW 528.64)
- Citrinin (*Penicillium citrinum*, CAS: 518-75-2, MW 324.28)
- FCCP (CAS: 370-86-5, MW 254.17)
- Potassium dichromate (CAS: 7778-50-9, MW 294.18)
- Ochratoxin A (*Aspergillus ochraceus*, CAS: 303-47-9, MW 403.81)
- Sterigmatocystin (CAS: 10048-13-2 MW 324.28)
- Triclosan (CAS: 3380-34-5, MW 289.54)
- Valinomycin (*Streptomyces* spp., CAS: 2001-95-8, MW 1111.32).

The toxic solutions were prepared from the dissolved toxins, serially diluted in ethanol, and screened for toxic responses in 10-fold dilutions. Thereafter, the end-points were determined using a series of two-fold dilutions. The tested concentrations ranged from 0.01 µg·mL^−1^ to 200 µg of ethanol-soluble substances per mL^−1^ cell suspension.

### 5.8. Cultivation, Toxicity Screening, and the Identification of Microbial Single Colonies

Airborne dust samples were collected from air filters, settled dust and building materials in buildings with environmentally compromised indoor environments. Single microbial colonies were observed four weeks after incubation, at room temperature, using the airborne dust samples cultivated on fungicide- and antibiotic-free tryptic soy agar (for bacteria) and malt extract agar (MEA) (for fungi), as described by Mikkola et al. [9]. Differentiation between fungal and bacterial colonies was verified by phase contrast microscopy.

The test procedure for microbial biomass suspended in ethanol has previously been described by Andersson et al. [5,30]. Briefly, ca. 10 µL of microbial biomass suspended in 200 µL of ethanol in a sealed ampule was heated in a water bath at 60 °C for 10 min. Using the spermatozoa motility inhibition assay, 200 µL of extended semen containing 5 × 10^6^ spermatozoa were exposed to 2 µL of biomass suspension. Using the cytotoxicity tests on somatic cell lines, ca. 2 × 10^4^ cells in 190 µL of medium were exposed in 10 µL of biomass suspension.

The toxic mold colonies were pure cultured on MEA and identified using ITS sequencing [5] as: *Acrostalagmus luteoalbus* (POB8, GenBank: KM853014.1); *Penicillium* *chrysogenum* (RUK/2, 100% identity with KU743900.1); *Aspergillus calidoustus* (MH34, GenBank: KM853016.1); *Trichoderma atroviride* (14/AM, GenBank: MH158554.1) [2]. Strain RcP61 was identified by sequence analysis of its calmodulin gene fragment [31] as *Penicillium expansum* (GenBank: KP889005.1). *Paecilomyces variotii* Paec 1, Paec 2, and *Aspergillus versicolor* SL/3 were identified at DSMZ (Braunschweig Germany).

## Figures and Tables

**Table 1:** EC_50_ concentration values obtained for the toxin-exposed spermatozoa after 20 min of exposure at 37 °C using two subjective spermatozoa motility inhibition assays involving visual inspection (VMI * and BSMI) and through the calculation of a CMI * index.

Toxins	EC_50_ (µg·mL^−1^)		
	CMI ^1^ (SD < 15%)	VMI ^1^	BSMI ^1^
Fungal toxins			
Chaetoglobosin A (*Chaetomium globosum*)	1	1–2	1–2
Alamethicin (*Trichoderma arundinaceum*)	5	3–5	5–10
**Bacterial toxins**			
Calcimycin (*Streptomyces* spp.)	0.4	1–2	1–2
Valinomycin (*Streptomyces* spp.)	0.0001	0.0003–0.0006	0.0003–0.0006
**Toxic chemicals**			
FCCP	1	0.5–1	0.5–1
Triclosan	1	0.5–1	0.5–1

* The CMI and VMI values were determined from the same video recordings (10× objective). The inhibition of boar spermatozoa motility was determined from the sample but estimated from video recordings using a different semen droplet (40× objective). ^1^ The median of three measurements, represented by four microscopic fields. The variation between measurements pertains to the two-fold dilution step.

**Table 2:** Toxic responses * identified using the SMID and CMI spermatozoa assays and the ICP assay.

Toxins	Response	EC_50_ (µg·mL^−1^)					Toxic Mechanism
		Motility Inhibition (Boar Spermatozoa)		ICP			
		CMI	SMID	FL	MNA	PK-15	
		20 min	2 h	1–3 d	1–2 d	1–3 d	
Fungal toxins							
Chaetoglobosin A (*Chaetomium globosum*)	I	1 ± 0.1	12 ± 2	1.3 ± 0.3	1.4 ± 0.2	2.7 ± 0.3	Inhibition of glucose transport ^1^
Alamethicin (*Trichoderma arundinaceum*)	III	5 ± 0.6	0.7 ± 0.1	11.5 ± 2	12 ± 4	10 ± 2	Potassium ion channel former ^2^
Sterigmatocystin (*Aspergillus* spp.)	IV	>20	>1000 ± 125	20 ± 5	7 ± 1	0.6 ± 0.1	Inhibitor of protein synthesis ^2^
Citrinin (*Penicillium citrinum*)	IV	>100	50 ± 20	104 ± 26		12 ± 5	
**Bacterial toxins**							
Calcimycin (*Streptomyces* spp.)	I	0.7 ± 0.3	>10 ± 1	1.4 ± 0.5	0.2 ± 0.06	0.4 ± 0.05	Calcium carrier ionophore and mitochondrial toxin ^3^
Valinomycin (*Streptomyces* spp.)	I	0.0001 ± 0.00002	70 ± 13	55 ± 16	1.7 ± 0.6	14 ± 4	Potassium carrier ionophore and mitochondrial toxin ^3^
Actinomycin D (*Streptomyces* spp.)	IV	>100	>100	<0.2	<0.2	<0.2	Inhibitor of RNA synthesis ^4^
**Toxic chemicals**							
FCCP	II	1 ± 0.1	2.7 ± 0.7	10 ± 2			Carrier protonophore ^5^
Triclosan	II	1 ± 0.1	2 ± 0.6	10 ± 2	4 ± 0.6	7 ± 2	Carrier protonophore ^5^
Potassium dichromate	IV	>200	>200	0.7 ± 0.3			
**Control**							
Ochratoxin A (fungal toxin from *Aspergillus* spp.)		>100	>100	>50	>50	>50	

* The test cells were exposed at 37 °C. ^1^ Nielsen & Frisvad [17]. ^2^ Bencsik et al. [1]. ^3^ Hoornstra et al. [18]. ^4^ Perry and Kelley [19]. ^5^ Ajao et al. [4].

## Supplementary Materials

The following are available online at http://www.mdpi.com/2072-6651/10/11/463/s1, Software Source Code S1: Semen Test Source Code.
