# Supplementary material for: An Evaluation of Boar Spermatozoa as a Biosensor for the Detection of Sublethal and Lethal Toxicity
Source: Toxins (Basel). 2018 Nov 8;10(11):463. doi: 10.3390/toxins10110463 (PMC6265928; doi:10.3390/toxins10110463)
Supplement: Supplementary file 1 [file toxins-10-00463-s001.pdf]

Supplementary Materials: An Evaluation of Boar Spermatozoa as a Biosensor for the Detection of Sublethal and Lethal Toxicity

Emmanuelle Castagnoli, Johanna Salo, Matti S. Toivonen, Tamás Marik, Raimo Mikkola, László Kredics, Alejandro Vicente-Carrillo, Szabolcs Nagy, Markus T. Andersson, Maria A. Andersson, Jarek Kurnitski and Heidi Salonen

SemenTest Source Code.

```
//  matstd.hpp
//
//
//  Created by Markus Andersson on 03/09/16.
//
//
// Copyright(C) 2016 Markus Andersson.
//
/*This file is part of SemenTest.

   SemenTest is free software: you can redistribute it and/or modify
   it under the terms of the GNU General Public License as published by
   the Free Software Foundation, either version 3 of the License, or
   (at your option) any later version.

   SemenTest is distributed in the hope that it will be useful,
   but WITHOUT ANY WARRANTY; without even the implied warranty of
   MERCHANTABILITY or FITNESS FOR A PARTICULAR PURPOSE.  See the
   GNU General Public License for more details.

   You should have received a copy of the GNU General Public License
   along with SemenTest.  If not, see <http://www.gnu.org/licenses/>.
*/
#ifndef matstd_hpp
#define matstd_hpp

#include <stdio.h>
#include <string>
#include <math.h>
#include <opencv2/highgui/highgui.hpp>
#include <opencv/cv.h>
#include <opencv2/opencv.hpp>
#include <iostream>
#include <string>
#include <sstream>
#include <fstream>
```

```
#if !defined(DEF_AM_OF_FRAMES)
#define DEF_AM_OF_FRAMES 100
#endif

using namespace std;
using namespace cv;

Mat mat_table(Mat I);
float matstd(Mat I);
float matmean(Mat I);
Mat normalizedImage(Mat I);
Mat differenceMatrice(Mat I, Mat II);
float analyseMobility(string file_name);
float mean_of_mobilityIndex(float* table, int size);
string fixDecimal(float mobility);
#endif /* matstd_hpp */

//
//  matstd.cpp
//
//
//  Created by Markus Andersson on 03/09/16.
//
//
// Copyright(C) 2016 Markus Andersson.
//
/*This file is part of SemenTest.

   SemenTest is free software: you can redistribute it and/or modify
   it under the terms of the GNU General Public License as published by
   the Free Software Foundation, either version 3 of the License, or
   (at your option) any later version.

   SemenTest is distributed in the hope that it will be useful,
   but WITHOUT ANY WARRANTY; without even the implied warranty of
   MERCHANTABILITY or FITNESS FOR A PARTICULAR PURPOSE.  See the
   GNU General Public License for more details.

   You should have received a copy of the GNU General Public License
   along with SemenTest.  If not, see <http://www.gnu.org/licenses/>.
*/

#include "matstd.hpp"

Mat mat_table(Mat I) {
        int channels;
        int rows;
        int columns;
        float intensityValue;
```

```
        Vec3b rgbVector;
        Mat intensityMatrix, calculation_matrix;
        //cout << "vidFeed: "<< I.rows << " " << I.cols << endl;
        //channels=I.channels();
        rows=I.rows;
        columns=I.cols;
        intensityMatrix.create(rows, columns, CV_32F);

        for (int i=0; i< rows; i++) {
                for (int j=0; j< columns; j++) {
                        rgbVector=I.at<Vec3b>(i,j);
                        intensityValue=((float)(rgbVector[0])+ (float)(rgbVector[1])+ (float)(rgbVector[2]));
                        intensityMatrix.at<float>(i,j)=intensityValue;
                }

        }

//   Mat calculation_matrix(1, (rows*columns), I.type());

/* Mat calculation_matrix, copyI;
//I.copyTo(copyI);

        if (intensityMatrix.isContinuous() == true) {
        calculation_matrix=intensityMatrix.reshape(1,1);
*/

        if (intensityMatrix.isContinuous() == true) {

                try {
                        calculation_matrix=intensityMatrix.reshape(1,1);
                        return calculation_matrix;
                } catch (...) {
                        cout << I.rows << " " << I.cols << endl;
                }
        } else {
                return I;
        }
        return I;
}

float matstd(Mat I) {
float std;
float entry;
float mean;

std=0.0;
entry=0.0;
```

```
mean=matmean(I);

/*

if (I.type()==sizeof(char)) {

for (int i=0; i<I.cols; i++) {
   entry=(double)I.at<char>(i);
   entry=entry-mean;
   entry=entry*entry;
   std+=entry;
}
} else if (I.type()==sizeof(int)) {
for (int i=0; i<I.cols; i++) {
   entry=(double)I.at<int>(1,i);
   entry=entry-mean;
   entry=entry*entry;
   std+=entry;
}
} else if (I.type()==sizeof(float)) {

for (int i=0; i<I.cols; i++) {
   entry=(double)I.at<float>(1,i);
   entry=entry-mean;
   entry=entry*entry;
   std+=entry;
}
} else {
*/
for (int i=0; i<I.cols; i++) {
   entry=I.at<float>(i);
   entry=entry-mean;
   entry=entry*entry;
   std+=entry;
}

//  } //jatka jos tarpeen!
std=std/(I.cols);
std=sqrt(std);

return std;
}

float matmean(Mat I) {
float sum;
float entry;

sum=0.0;
entry=0.0;
```

```
//cout << "matmean columns: " << I.cols << endl;
//cout<<"I type: "<< I.type()<<endl;
//cout<<"size of double: "<<sizeof(long double)<<endl;

/*
 if (I.type()==sizeof(char)) {

for (int i=0; i<I.cols; i++) {
entry=(double)I.at<uchar>(0,i);
//cout<<I.at<char>(0,i)<<endl;
sum+=entry;
}
} else if (I.type()==sizeof(int)) {

for (int i=0; i<I.cols; i++) {
   entry=(double)I.at<int>(0,i);
   //cout<<I.at<char>(0,i)<<endl;
   sum+=entry;
}
} else if (I.type()==sizeof(float)) {

   for (int i=0; i<I.cols; i++) {
         entry=(double)I.at<float>(i);
         //cout<<I.at<float>(i)<<endl;
         sum+=entry;
   }
} else {
*/

for (int i=0; i<I.cols; i++) {
   entry=I.at<float>(i);
   //cout<<I.at<double>(i)<<" ; " << entry << endl;

   sum+=entry;
}
//  } //jatka jos tarpeen!
//cout << "sum of mean: " << sum << endl;
sum=sum/(I.cols);
// cout << "sum of mean: " << sum << endl;
return sum;
}

Mat normalizedImage(Mat I) {

float entry=0.0;
float mean, standard_dev;
Mat newMatrice;
```

```
newMatrice=Mat::zeros(I.rows, I.cols, CV_32F);


mean=matmean(I);
standard_dev=matstd(I); //standardeviation.

for (int i=0; i<I.cols; i++) {
   entry=I.at<float>(i);

   entry=entry-mean;
   entry=entry/standard_dev;
         //cout << entry << endl;
   newMatrice.at<float>(i)=entry;
         //cout << newMatrice.at<float>(i) << endl;
}

return newMatrice;
}

Mat differenceMatrice(Mat I, Mat II) {
         Mat III;
         III=Mat::zeros(I.rows, I.cols, CV_32F);
         if (I.size()==II.size() && I.channels() == II.channels()) {
         subtract(I, II, III);
         } else {
                  cout << "ERROR." <<endl;
                  III.eye(2, 2, CV_32F);
         }
         return III;
}

float mean_of_mobilityIndex(float table[], int size) {
         float sum=0;


         for (int i=0; i < size; i++) {
                  sum=sum+(table[i]);

         }
         sum=sum/(size);

         return sum;
}

float analyseMobility(string file_name) {
  //default capture width and height
         int k=0, counter=0, mobility_index_length;
         float mobility=0.0, average_mobility;
         float mobility_index[DEF_AM_OF_FRAMES];
```

```
    VideoCapture capture(file_name);
    Mat vidFeed, muunnos, normalisoituMat, refMatriisi, diffMatriisi, absdiff_mat;

    if (capture.isOpened()) {
        capture.read(vidFeed);

            muunnos=mat_table(vidFeed);
            normalisoituMat=normalizedImage(muunnos);
            refMatriisi=normalizedImage(muunnos);

        while ((counter < DEF_AM_OF_FRAMES) && capture.read(vidFeed) && (vidFeed.cols > 0) ) { //Store
image to matrix named vidFeed.

                //capture.read(vidFeed);
            muunnos=mat_table(vidFeed);
                //cout << matmean(muunnos) << endl;
         normalisoituMat=normalizedImage(muunnos);
                //cout << matmean(refMatriisi) << endl;
                diffMatriisi=differenceMatrice(normalisoituMat, refMatriisi);
                absdiff_mat=abs(diffMatriisi);
                mobility=matmean(absdiff_mat);
                mobility_index[counter]=mobility;
                //cout << mobility << endl;
                //refMatriisi.deallocate();
                refMatriisi=normalizedImage(muunnos);
                //if (k<2) {
                        //cout << matmean() << endl;
                        //k++;
                //}
                counter++;

        }


                capture.release(); //Closes video file. May cause problems if the file is already closed
automatically!
            mobility_index_length=counter;
            //cout <<" Length of array: " << mobility_index_length << endl;
            average_mobility=mean_of_mobilityIndex(mobility_index, mobility_index_length);
            return average_mobility;
            } else {

            cout<<"Video file won't open!"<<endl;
                throw 20;
                return 1;
    }
}
```

```
string fixDecimal(float mobility) {
        string fixed_number;

        fixed_number=to_string(mobility);
        for (int i=0; i < fixed_number.size(); i++) {
                if (fixed_number[i]== '.') {
                        fixed_number[i]=',';
                }
        }
        return fixed_number;
}

//
//  SemenTest.hpp
//
//
//  Created by Markus Andersson on 03/09/16.
//
// Copyright(C) 2016 Markus Andersson.
//
/*This file is part of SemenTest.

   SemenTest is free software: you can redistribute it and/or modify
   it under the terms of the GNU General Public License as published by
   the Free Software Foundation, either version 3 of the License, or
   (at your option) any later version.

   SemenTest is distributed in the hope that it will be useful,
   but WITHOUT ANY WARRANTY; without even the implied warranty of
   MERCHANTABILITY or FITNESS FOR A PARTICULAR PURPOSE.  See the
   GNU General Public License for more details.

   You should have received a copy of the GNU General Public License
   along with SemenTest.  If not, see <http://www.gnu.org/licenses/>.
*/

//

#ifndef SemenTest_hpp
#define SemenTest_hpp

#include "matstd.hpp"

float CMI_as_percentOfControll(float MI_experiment, float MI_background, float MI_control);

#endif /* SemenTest_hpp */


//
```

```
//  SemenTest.cpp
//
//
//  Created by Markus Andersson on 03/09/16.
//
// Copyright(C) 2016 Markus Andersson.
//
/*This file is part of SemenTest.

   SemenTest is free software: you can redistribute it and/or modify
   it under the terms of the GNU General Public License as published by
   the Free Software Foundation, either version 3 of the License, or
   (at your option) any later version.

   SemenTest is distributed in the hope that it will be useful,
   but WITHOUT ANY WARRANTY; without even the implied warranty of
   MERCHANTABILITY or FITNESS FOR A PARTICULAR PURPOSE.  See the
   GNU General Public License for more details.

   You should have received a copy of the GNU General Public License
   along with SemenTest.  If not, see <http://www.gnu.org/licenses/>.
*/
//

#include "SemenTest.hpp"

float CMI_as_percentOfControll(float MI_experiment, float MI_background, float MI_control) {
//background sample is first parameter, control sample second and after that starts the series of the real samples.
	float result=0.0;
	if (MI_control > MI_background+ 0.000001 || (MI_control < MI_background - 0.000001)) {
	result=(MI_experiment - MI_background)/(MI_control - MI_background);
	result=result*100;
	} else {
		throw "Back ground sample mobility and control sample mobility too similar!";
	}
	return result;

}

int main(int argc, char* argv[]) {
	Mat I, muunnos;
	float tulos;
	float mobility_of_sample[13];
	string mobility_number_fixed="";

	cout << " SemenTest  Copyright (C) 2017  Markus Andersson
   This program comes with ABSOLUTELY NO WARRANTY; for details, see GNU General Public License at <http://www.gnu.org/licenses/> "  <<endl;
```

```
    if (argc < 2) {
            cout << "No file submitted as parameter." << endl;
            return 1;
        }

        for (int i=1; i< 13; i++) {
            try {
                if (i < argc) {
                    tulos=analyseMobility(argv[i]);
                    mobility_of_sample[(i-1)]=tulos;
                } else {
                    mobility_of_sample[(i-1)]=0;
                }
            } catch (int e) {
                cout << "Error in opening video file." << endl;
                return 1;
            }
            catch (...) {
                cout << "Some other error than problem with video file." << endl;
                return 1;
            }
        }

            ofstream result_file;
            result_file.open("C:/SemenTest_app/SemenSampleMobilities.csv");

            for (int j=2; j< (argc-1); j++) {
                try {


        mobility_number_fixed=fixDecimal(CMI_as_percentOfControll(mobility_of_sample[j],
mobility_of_sample[0], mobility_of_sample[1]));

                result_file << "Sample "<< (j-1) << ";" << mobility_number_fixed << endl;
                } catch (const char* msg) {
                    cout << msg <<endl;
                    break;
                }
            }
            result_file.close();


        waitKey(10);

  return 0;

}
```
